# A dialogue-based web application enhances personalized access to healthcare professionals – an intervention study

**DOI:** 10.1186/1472-6947-12-96

**Published:** 2012-09-04

**Authors:** Charlotte D Bjoernes, Birgitte S Laursen, Charlotte Delmar, Elizabeth Cummings, Christian Nøhr

**Affiliations:** 1 Department of Development and Planning, Danish Centre for Health Informatics, Aalborg University, Vestre Havnepromenade 5 1. sal, DK-9000, Aalborg, Denmark; 2 The Clinical Nursing Research Unit, Aalborg Hospital, Aarhus University Hospital, Aarhus, Denmark; 3 Department of Public Health, Aarhus University, Aarhus, Denmark; 4 Health Services Research Group, University of Tasmania, Tasmania, CIS, Australia; 5 Department of Development and Planning, Danish Centre for Health Informatics, Aalborg University, Aalborg, Denmark

**Keywords:** Health informatics, Internet communication, Prostate cancer, Short stay patients, Empowerment

## Abstract

**Background:**

In today’s short stay hospital settings the contact time for patients is reduced. However, it seems to be more important for the patients that the healthcare professionals are easy to get in contact with during the whole course of treatment, and to have the opportunity to exchange information, as a basis for obtaining individualized information and support. Therefore, the aim was to explore the ability of a dialogue-based application to contribute to accessibility of the healthcare professionals and exchangeability of information.

**Method:**

An application for online written and asynchronous contacts was developed, implemented in clinical practice, and evaluated. The qualitative effect of the online contact was explored using a Web-based survey comprised of open-ended questions.

**Results:**

Patients valued the online contacts and experienced feelings of partnership in dialogue, in a flexible and calm environment, which supported their ability to be active partners and feelings of freedom and security.

**Conclusion:**

The online asynchronous written environment can contribute to accessibility and exchangeability, and add new possibilities for dialogues from which the patients can benefit. The individualized information obtained via online contact empowers the patients. The Internet-based contacts are a way to differentiate and expand the possibilities for contacts outside the few scheduled face-to-face hospital contacts.

## Background

This paper reports the qualitative effects from the patient users’ perspective of a dialogue-based application specifically designed to accommodate their information and communication needs.

An intervention study was undertaken to design and implement a health informatics tool including dialogue-based software to establish asynchronous written dialogue between the individual patient at home and healthcare professionals from the short stay hospital setting. This group of healthcare professionals is involved in the patient’s care and is well-known to the patient.

Today’s healthcare systems are characterised by short stays in hospital with planned discharge typically occurring within one or two days of surgery, even after large operations. This is exemplified by focusing on men with prostate cancer in a surgical course of treatment: A radical prostatectomy to remove the whole prostate gland, including the tumour, and thereby to cure the cancer. The typical experience for these men is a single hospital admission, from the day of surgery to discharge occurring one or two days after the surgery, a maximum of 3 days. This is then supported by 14-16 short scheduled contacts in outpatient clinics before or after the surgery. So it can be seen that the course of treatment and care is based upon short and scattered interactions that limit the time and opportunity for contact between the patients and the healthcare professionals.

According to satisfaction surveys patients are satisfied with shorter stays in hospital in general [1-3]. Discharge within one day confirms the quality of the treatment and the patients expect to be cured without complications. However, the patients need to learn to cope and live in this new and unknown life situation, e.g. how to handle the risk for long-term side effects such as incontinence. The healthcare professionals often play a significant role in this process of learning [4-8], but due to the reduced admission time at the hospital, the patients have to learn quickly. The short hospital stays have the effect that the patients’ concerns and information needs must be focused and timely. To find out what the individual patient’s needs are the dialogues are essential [9]. The dialogues help the healthcare professionals to recognize what is important for this unique and individual patient and thereby to act upon and accommodate these individual requests or requirements.

However, due to the short hospital stays the actual time for dialogues is reduced. In other words, the restricted time leaves very little time to find out what information the individual patient in fact requires and there is no room for the patients’ individual needs. Thereby, the short contacts reduce the patients’ time for individualized information and support [10]. However, according to a survey of the literature, it does not appear to be the amount of time that is important for the patients rather it is the ability to get in contact, and the quality of these contacts. The literature survey identified that men with prostate cancer, treated with prostatectomy surgery, often do not experience the healthcare professionals at the hospital as being available to their needs [11]. Harden et al. [12] explain how the patients experience the healthcare professionals as very busy, which prevents the patients asking questions. Moore and Estey [13] describe how the men feel self-conscious and uncomfortable contacting the healthcare professionals, and therefore avoid doing so, even when they consider that their concerns are very important. In the study by Hedestig et al. [14] the men problematized the time at home between their checkups at the hospitals. The men explain how they often had difficulties contacting the healthcare professionals or getting answers out of them. In the study by Harden et al. [12] the men give details of how they function well most of the time, however, there was still ten percent of the time that was difficult. When these relatively demarcated concerns were not addressed the issues often got out of proportion and became serious concerns.

Harden et al. [12] found that the men thought it would be valuable for them to have a person to contact. According to Moore and Sherwin [15] being able to access expert information and advice, in times of need and in between meetings at the hospital, is in generally important to patients. Milne et al. [16] through their description of how the accessibility of the healthcare professionals is important conclude that being at home without readily available support and advice causes anxiety and uncertainty. This illustrates how healthcare professionals must be available for contact by patients throughout the patients’ course of treatment and care. It is essential that the patients experience the healthcare professionals as easy to get in contact with both at the hospital and when the patients are home.

**Figure 1:**
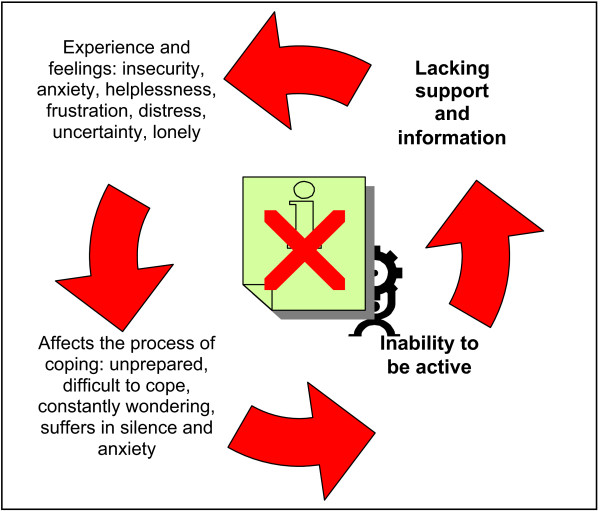
**The process of disempowerment caused by the lack of individualized information and support**.

The literature survey demonstrated that patients often do not receive the individualized support, information, and dialogue that they need, and this can lead to feelings of insecurity and uncertainty [16,17]. This process of disempowerment caused by the lack of individualized information and support is depicted in Figure 1. The figure provides a summation derived from the findings of the survey of the literature. The terms uncertainty and insecurity are closely related: Uncertainty is primarily attributed to the sensation of feeling unsure about the best action or choice in a given situation; insecurity is to feel unsafe or even uncertain; a state of doubt about the future or about what is the right thing to do. Thus, the negative effects of being insecure or uncertain point to the same end-point: Disempowerment, which is a feeling of powerlessness or helplessness that reduces the amount of control that someone, has over a situation. Thereby, Figure 1 gives a picture of the importance of gaining individualized information and support in a course of treatment and care. Hence, by providing information and support healthcare professionals may be able to empower the patients. Empowerment is the process of giving somebody power in a particular situation [11], e.g. to give the individual patient more control over their own life or the situation they are in by individualising the information, as different patients need different information, at different times, and in different ways.

The dialogues are essential to individualize the information and support, but the dialogues between the individual patient and the healthcare professionals presuppose two aspects. First, availability, meaning the accessibility of the healthcare professionals: it must be easy for the patients to get in contact with the healthcare professionals during the whole course of treatment. Secondly, there must be a “room” for exchanging: information, experiences, resources, questions, and answers, as these exchanges are essential for individualizing the information and support. A form of: Exchangeability, as both the healthcare professional and the patient may be able – and may have the opportunity – to interchange conceptions.

Thus, there is a contradiction between the patients’ needs for contact and the short stays at hospital with few specified and scheduled contacts between the patients and healthcare professionals. However, asynchronous written environments, which the Internet offers, could be a possible way to differentiate when informing the patients and thereby to expand the possibilities in the contacts outside the few formal face-to-face contacts at the hospital.

In sum, the survey of the literature initiated the idea of using the flexibility the asynchronous dialogue-based Internet technologies offer to support patients, as these websites allows the users to communicate without being present, at the same time. The use of an asynchronous environment could be a way to establish availability whilst at the same time the written environment could be a way to establish “a room for” exchangeability. Therefore, an application was developed and implemented in clinical practice, and subsequently the qualitative effects were explored.

## Method

Considering patients’ short stays in hospital a new patients’ health informatics tool was developed: The Online Patient Book^©^. This tool was designed to meet the information and communication needs of a specified group of patients: Men with prostate cancer treated with surgery, in a short stay hospital setting; a specified urology department in Denmark. A central application integrated into this health informatics tool was an application for asynchronous, written dialogue between the users according to the findings in the survey of the literature. Such applications provide the opportunity for an ongoing connection, and thereby contact between the individual patient and healthcare professionals.

**Figure 2:**
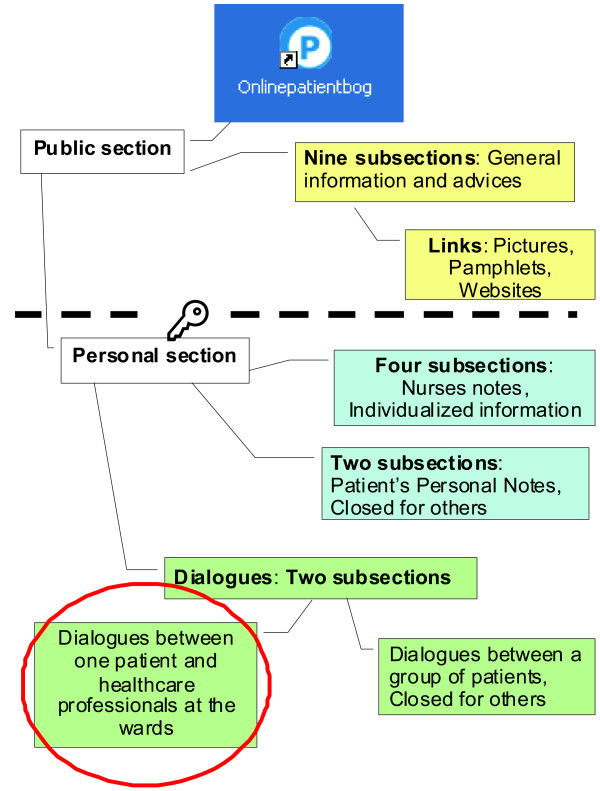
**The different applications available as part of the Online Patient Book.**^**©**^

Figure 2 provides an overview of the different applications available as part of the Online Patient Book^©^. The website consists of two sections, each with further subsections: A public section, with open access, that provides monologue-based general information generated by clinical experts. The public section is supplemented with a secure individualized section that requires a login. This personal section provides monologue-based individual personalized information, which can be generated both by healthcare professionals and by the patient himself. Additionally, it provides functionality that facilitates personal communication between the individual patient and healthcare professionals. The Online Patient Book^©^ conveys a link to the healthcare professionals at the particular hospital wards the patients consult during the actually course of treatment. The Online Patient Book^©^ also contains an application for patient-to-patient dialogue, with each patient user being connected to a small group of other patient users. Thus, the patients are offered the opportunity to engage in these two different types of dialogues within this asynchronous and written environment.

### Aim

This paper focuses on the evaluation of the effects of the dialogue-based application between the individual patient and the well-known healthcare professionals. This was undertaken to explore whether, and how, this central application is valuable in relation to the importance of accessibility of the healthcare professionals and exchangeability between the two partners. The question asked in the evaluation was:

"How can an online contact contribute in the overall contact between men with prostate cancer and the healthcare professionals?"

This question was relevant to get insight and a deeper understanding of the potential of Internet based dialogues in short stay hospital treatment settings.

### Ethics

In the overall project the generating, handling, and publication of data are consistent with the guidelines of CVK (The Danish research ethics committees) and reported under Datatilsynet (Danish Data Protection Agency) (2008-03-05). The patient users’ descriptions and statements, which were conducted in the evaluation, have been translated from Danish omitting any corrections in phrasing. Additionally, this research adheres to the RATS guidelines on qualitative research.

The Online Patient Book^©^ is established under the standard security approval and procedures at the IT Department, The North Denmark Region, Denmark. The public section of the Online Patient Book^©^ is open for all Internet users via the World Wide Web address. Access to the personal section of the Online Patient Book^©^ requires a key code. The healthcare professionals use their personal standard id and key code to the hospital’s general IT systems. The patient users log on by their social security number and a personalized auto-generated key code. The nurses in clinical practice handled the enrolment of individual patients via a specified web page in the Online Patient Book^©^. The enrolment automatically generated an e-mail to the patients private e-mail box containing system generated, secure one way encryption key codes. This encryption is considered compliant with Danish safety and security legislation. Due to the security of the system, the patient users could not change the personal key code.

### Intervention

The research was conducted as an intervention study, meaning [18-21]: From an experienced and substantiated problem to use and evaluate the effects of a tool in clinical practice. The Online Patient Book^©^ was implemented in clinical practice in September 2009 in the Department of Urology, Aalborg Hospital, Aarhus University Hospital, Denmark. It was introduced as a new information and communication tool for men with prostate cancer who were starting their course of treatment and planning for prostatectomy surgery. The nurses on the wards handled the Online Patient Book^©^, as an element of the standard care provided to this group of patients. Once registered the patient users could use the tool during their whole course of treatment, and thereby both before and after their surgery.

### Evaluation

**Figure 3:**
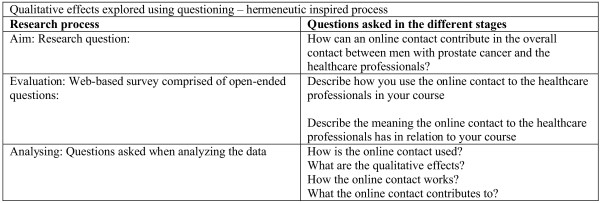
Qualitative effects explored using questioning – hermeneutic inspired process.

The intervention was followed by an evaluation of the qualitative effects of the intervention from the patient users’ perspectives. As the aim of the evaluation was to generate insights in the online, asynchronous and written environment’s ability to establish a valuable contact between the individual patient and the healthcare professionals the evaluation method was inspired by the hermeneutic philosophy [22,23]. In the hermeneutic perspective questioning is a core aspect to get insights and understandings. Figure 3 documents the questions asked in the different stages of the research process: From the research question to the questions asked in the process of analysing the data from the evaluation.

A Web-based survey, consisting of a sequence of open-ended questions, was conducted via a webpage in the Online Patient Book^©^[24]. The patient users answered the open-ended questions by describing their use of, and what they thought about, the tool in textboxes without word limitations. Examples of the open-ended questions are provided in Figure 3. To minimize possible bias, the patient users were informed that the healthcare professional users would not have access to the evaluation section in the Online Patient Book^©^ or their answers. This was of concern as the patient users and the healthcare professional users were known to each other and the patient users were still engaged in the course of their treatment. Therefore, it was considered possible that if the healthcare professional users could read the patients’ evaluation it may influence the patient users’ responses.

The evaluation was carried out from June 2010 until September 2010. Patient users were invited to evaluate the system from two to seven months after their surgery. As the evaluation ceased in September 2010 patient users who had their surgery after July 1st 2010 were excluded from the evaluation. This resulted in a total of 58 potential evaluators, of whom 34 completed and sent the written responses; response rate 59%. Thus, the results are based on data from the 34 written responses received.

### Analysis

**Figure 4:**
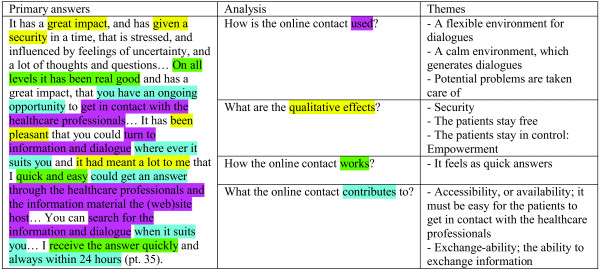
Example of how the process of hermeneutic questioning generated core themes.

To explore the patients’ experiences of their online contacts with the healthcare professionals and what it meant to them to have this particular application the analysis of the data was based on hermeneutical interpretation of meaning [24]. The hermeneutic approach was used to explore the patients’ answers by entering into a dialogue with the evaluation-text. The patient users’ evaluations were handled as primary answers to the questions asked in the evaluation. In the process of analysis, new questions were asked to continue the (hermeneutic) questioning. The questions asked in this process of analysis are listed as the third stage in the Figure 3. What the primary answers stated about the use and effects of the online contact were identified and understood by a continuous back-and-forth process between the different levels of questions and the primary answers. Although it is difficult to depict the circularity between answers and questions, and between a description from one patient user and the whole data text Figure 4 provides an example of how the process of questioning generated core themes. As such, this circularity generated core themes that relate to the application of asynchronous, written contact between the individual patient and the group of healthcare professionals at the specified department.

**Figure 5:**
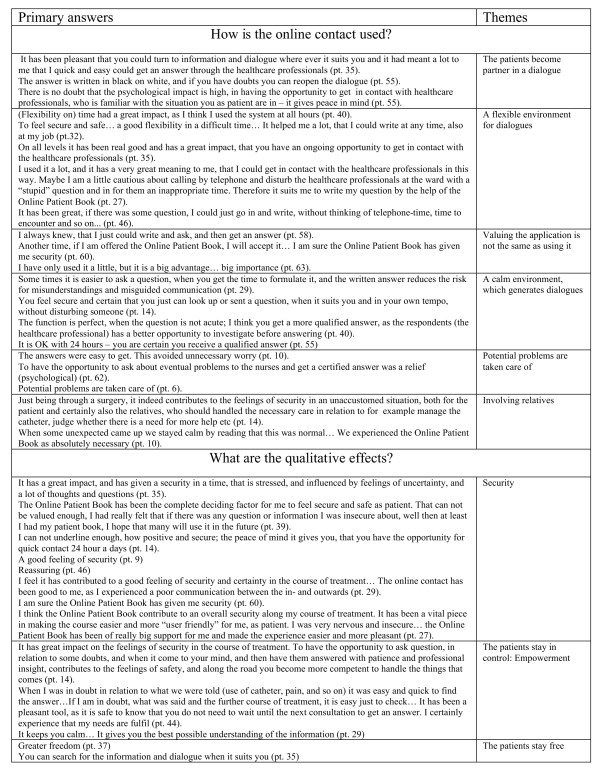
Documents the analysis of data: hermeneutical interpretation of meaning: from primary answers to themes.

Figure 5 documents the analysis of the data. The heading in the figure: Primary answers, lists key quotes that represent the core themes provided in relation to the application for dialogue between the individual patient and the healthcare professionals. Figure 5 thereby forms the basis for the results presented in the following section of this paper. For the purposes of this paper, the key quotes are translated versions of the men’s written responses provided in the Web-based survey, which were completed in Danish. During the analysis stage the data was used in its native language. The translation was discussed across the board of authors to omit any corrections in phrasing from the primary written answers.

## Results

The results are presented under the four questions asked in the process of analysing, and thereby the sum of core themes provided in relation to the web application for dialogue between the individual patient and the healthcare professionals. As listed in Figure 5 a total of fourteen themes were identified: Six themes relate to the use of the online contact; four themes explaining the effects of the online contact; one theme pointing to how the online contact worked; and finally three themes summing up the background for the effects by focusing on what the online contact contributes to.

### How is the online contact used?

The way the patients explain how they use the online contact illustrates how they experience the opportunity to get in contact with the healthcare professionals as being a partner in a dialogue. The patients themselves use the term dialogue when they describe their use of the application. At the same time the primary answers illustrate that it means a lot that the Online Patient Book^©^ is a link to the departments the patients consult during their course of treatment. The patients stress the advantages in the fact that the healthcare professionals know who they are, because they are patients in their department. Although, it is important to be aware of the duality inherent in these expressions as the patients actually point to the importance for them of being familiar with the healthcare professionals. It seems that just by linking to the department the patients are familiar with, the patients feel closer to the healthcare professionals, even though they may not have meet the actual healthcare professionals who answer their inquiry.

The flexibility of the asynchronous environment, which the online contacts are based on, seems to be one of the important advantages of the Online Patient Book^©^. Nearly every patient user commented positively about this flexibility using words as: That was pleasant, VERY great impact, very important, nice, it was super, real good, most important, great impact, just ideal – perfect, it has been good. Several patients described how they used the Online Patient Book^©^ at very different hours. Patient users declare how it was important for them to have this opportunity as it helps them moving on with their normal life. Additionally, the men get the feeling that they are not disturbing the healthcare professionals, as they would if they use the telephone. However, it is also reasonable to assume that some problems, for example after surgery, will be handled before they progress to the extent that they become complications that demand readmission of the patient. Patient users described how they used the opportunity to ask questions in relation to potential problems. One patient actually described that potential problems are taken care of through the use of the Online Patient Book^©^.

It appears that the patients experience the online asynchronous environment as a calm environment, which gives them more time to both formulate their own questions and also to understand the answers received. The patients even identified some advantages in not receiving the answer from the healthcare professionals right away. One patient pointed to more qualified answers being received from the healthcare professionals through this method, as the healthcare professionals are less likely to be interrupted in the same matter, as for example when the patients call by phone.

Analysing the evaluation text illustrated that even though the patients emphasize the importance of an application it does not necessarily mean that they use it. In relation to the opportunity to get in contact with the healthcare professionals it appears that the possibility in it self contributed to a feeling of security. This indicates that valuing the application is not the same as using it.

The patient users often used the term ‘we’, when they explain their use of the online contact. The use of this term depicts how their relatives were often involved in the use of the tool. This is underlined by concrete explanations on how the online contacts also were used by, and of value to, the relatives.

### What are the qualitative effects?

Responses from patient users indicated that for many of them, the Online Patient Book^©^ has been of value, and for several very important, to their feelings of security during their course of treatment and care. The majority, 33 of the 34 patient users, valued the opportunity to get in contact with the healthcare professionals and considered this positively. Verbs used to describe the value of the dialogue-based application ranged from: practical and useful to; all-important and having a very great impact. One patient was neutral in his evaluation of the dialogue-based application, however he still used the opportunity to contact the healthcare professionals via the Online Patient Book^©^. He explains how he utilized the freedom the asynchronous contacts offer, but he also liked the opportunity to speak directly to the healthcare professionals by phone.

All of the patient users who provided evaluations emphasized the tools flexibility. Several patients articulated the feeling of freedom resulting from having access to the tool. As such, it appears that simply by having a tool like the Online Patient Book^©^ the patients experience a kind of freedom. Some of the patient users relate the freedom to a feeling of security. The asynchrony and flexible environment that the Online Patient Book^©^ is based on is thereby one of the significant advantages of the tool. The online connection to the hospital is recognised as an open door the patient can use when he has the time and need, without interrupting his personal day of life. Therefore it appears to be important to offer a tool the patient can use whilst not being bound by time and place.

The patients seem to have been empowered by having the Online Patient Book^©^ as their tool during the course of their treatment and care. As a patient user made clear, they use the tool to be informed of what will occur ahead of new challenges and through that maintain some control, in a situation that is new and at times unpredictable. Being empowered is important, as the patients struggle with the new and unknown life situation they need to find out about. The patient’s own body can get out of control and some patient users explained how they used the Online Patient Book^©^ to be at the front of these bodily problems, and by that still stay in control.

The patient users’ descriptions do not only illustrate that they use the Online Patient Book^©^ as a tool, but also that the patients recognize it as a tool. One patient calls it “a pleasant tool”. The tool is their tool, as it fits in their hand like a hammer and helps them to move on. By using the tool, the men stay in control. From a phenomenology perspective such a distinction is relevant, as the significance of tools arise and are experienced in our “life-world”. There is a distinction between various types of experiences from thought, imagination, bodily awareness, and embodied action - as experienced from the subjective or first person point of view [25].

In addition, it seems that the patient users especially benefited from the calmness the asynchronous environment contributed to. The patient users could both take as long as they needed to ask the “right” question, but also do so without feeling the stress in relation to taking someone’s time. The patients stay in control and feel they are competent and so become empowered partners in the dialogue.

The descriptions provided by the patient users of how potential problems are taken care of points to how prioritizing the development of the patients’ health informatics tool could ultimately save resources in the long term. The patient users believed that providing online contact lowered the resource consumption by reducing the time spent on issues for both the patients and the healthcare professionals. In that way the patient users’ responses suggest evidence of potential additional quantitative effects.

### How the online contact works?

The application for providing asynchronous written dialogue between the single patient and the healthcare professionals was established on a 24 hours response time. Thus the patient user was promised, and could require, a response from the healthcare professionals within 24 hours. The findings in the evaluation substantiated how this time interval was central to the positive effect of the online contact. The primary answers to this question depict evidence that the 24-hour responses were experienced as fast responses and this contributed to a sense of an ongoing dialogue. Few patient users stressed the importance of the 24 hours answer, by complement their evaluations with a: Thank you. The patients thereby confirm that they had the answer within 24 hours and that this was very important for them. Thus, it seems that the 24-hour administration, seven days a week, is vital for the online contact to be of value.

### What the online contact contributes to?

The patient users’ descriptions illustrate that they found it easy to get in contact with the healthcare professionals by utilizing the online contact in the Online Patient Book^©^. Thereby, the health informatics tool contributes to the accessibility of the healthcare professionals and establishes a stable, ongoing connection to the healthcare professionals at the specified departments throughout the course of treatment and care.

The way the patients describe their use of the online contact illustrated how they exchange information, questions, and answers with the healthcare professionals by utilizing the online contact. As such, the Online Patient Book^©^ seems to contribute to an exchangeability, as both the healthcare professional and the patient have the opportunity to interchange conceptions.

As described above, these two aspects are the basis for establishing a dialogue between the individual patient and the healthcare professionals – a dialogue that is essential to individualize the information and support. Firstly, the availability or accessibility of the healthcare professionals; it must be easy for the patients to get in contact with the healthcare professionals during the whole course of treatment. Secondly, there must be a “room” for exchanging; information, experiences, resources, questions, and answers, as these exchanges are essential to find out, what the individual patient’s in fact requires in relation to information and support.

As described earlier the availability of the healthcare professionals and the room for exchanging information is restricted in short stay hospital settings, which in turn reduces the patients’ opportunity for obtaining individualized information. Thus, the online contact appears to add new possibilities to determine what information the individual patient actually requires, as the healthcare professionals are available for the patient outside the short stay, and as the patients as well as the healthcare professionals are offered a room to exchange information, experiences, resources, questions, and answers. The dialogue-based Internet technology offers the opportunity for patients and healthcare professionals to remain in contact and to exchange information. The patients experience the healthcare professionals as easy to contact and as available 24 hours a day via the online room. At the same time the patient users descriptions substantiated that the quality of these contacts are good. Some patients even characterize it as their life-line.

The asynchronous written environment seems to offers a room that differs from other rooms for contact. Firstly, the patient users of the Online Patient Book^©^ managed to utilize the flexibility and calmness the online contact offers. The patients emphasize the feelings of having their freedom from the hospital system whilst at the same time feeling secure, as they know that the healthcare professionals are only a move, on their own computer, away from them and at all times. The patients can access expert information and advice in times of need, which is important to patients. The asynchronous written environment, as offered by the Internet, is a way to expand the possibilities in the contacts outside the few formal face-to-face contacts at the hospital.

**Figure 6:**
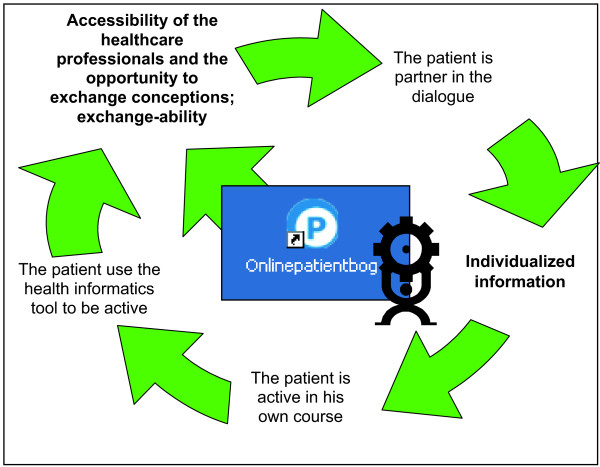
The individualized information obtained via online contact empowers the patients.

The patients therefore experience the online contact as that of being a partner in a dialogue, they act as active participants in the dialogue, and they use the health informatics tool to be active, this is depicted in Figure 6. By adding online contact to the restricted meetings at the hospital the healthcare professionals have the opportunity to accommodate the patients need for different information, at different times, and in different ways. In other words, the healthcare professionals can provide individualized information and support and thereby initiated the patients’ process of empowerment. It is not possible to make the patient’s essential learning process more efficient, but through this tool it is possible to expand the time available for the learning process to occur.

Secondly, words and phrases such as: a tool; a lifeline; open 24 hour; the hospital is right next to you, illustrate how this application for written, asynchronous dialogue is different from a general E-mail dialogue. When the patients send a request their experience is not that they are distributing a letter to a large mailbox at the hospital. Instead, it seems that the patients experience the direct link to the healthcare professionals as a network group. The patients’ descriptions illustrate, how they experience a shared room, to which they can open the door (the tool) and enter it at any time. In the room they are part of a familiar network and partner in a dialogue. The dialogue is regularly on standby, however ready to be open again at any time if new doubts arise. The shared information is easy to find, check, and re-read as the written dialogue is saved in chronological order by dates. The patients can easily find answers that were given a month ago, which can be difficult if the patients get the answers to their general private E-mail box, that also keeps E-mails e.g. from their friends, flight tickets, and news feeds.

As a core intention here was to accommodate the patients’ need for access to of the healthcare professionals during the patients’ course of treatment and care, it is essential that the patients actually had some experience of the healthcare professionals being easy to access. The patients use the health informatics tools because they need it in their difficult and insecure situation. The asynchronous environment contributes to a kind of freedom, as the health care system in some way always seems to offer an open door. The patients can use – enter – the door when they need it and in their own tempo. Thereby, the health informatics tool contributes to the accessibility of the healthcare professionals, and establishes stability and connection throughout the course of treatment and care. To patients the flexibility is a very important single reason to use the Internet, as it offers a dialogue that is not bound by time and place. It appears that when patients have this tool; the Online Patient Book^©^, in their hand the time spend on “just” being a patient is reduced. This is important, as the individual can continue his normal day of life and as it is evident that when patients are active the risk for complications after surgery, are minimized [26].

## Discussion

The analysis of the results reveals that the majority of patients could benefit from the use of health informatics tools as the Online Patient Book^©^. The majority who evaluated their use of the Online Patient Book^©^, 32 out of the 34 patient users, stated that the tool contributed positively to their feelings of security and certainty during their course of treatment and care. The two patient users who described that the tool had no effect on their feelings of security or certainty still indicated that they valued or utilized the freedom to use the tool when they needed and explained how the tool helped them maintain their overview during their course of treatment. None of the completed responses contained descriptions that pointed directly at negative effects from the use of the tool. This points to the fact that the tool in general empowered the patient users. However, it is important to consider that a higher number of the patients who valued the Online Patient Book^©^ in their course of treatment and care responded to the invitation to evaluate the system than those who did not use or value the tool.

For the purpose of this evaluation, in combination with a population who already were familiar with this particular web environment, the Web-based survey via the Online Patient Book^©^ was a relevant and appropriate evaluation mechanism. Although, it is acknowledged that face-to-face interviews may potentially contribute to more in-depth insights than those depicted in written evaluations. This is due to the more cognitive demanding task of writing the answers rather than responding to verbal questions [27-30]. However, from a qualitative perspective the patients indicated that the Online Patient Book^©^ was important. Interestingly, this was confirmed in a general hospital patient satisfaction survey conducted in 2010 by Ruby and Poulsen [31]. Two of the Online Patient Book^©^ users were informants in this face-to-face interview study and the interviewer was unaware of the Online Patient Book^©^. Even so the two informants spontaneously talked about the basic idea in this tool [31]:

"It (the Online Patient Book^©^) has especially impressed me very much…It is really good, if you just have some questions or something you just have a little doubt about. And for me it is effective, as if I wake up at 3 a.m., I can write, and they (the healthcare professionals) can answer, when they have the time (page 38)."

These external findings illustrate the potential of integrating Internet technologies, and especially utilising the online, asynchronous and written environment, in the future patient information.

The findings of this research confirm the conclusion of Pinnock et al. [32] in relation to their project which developed a website for men with prostate cancer in general. Using the early Internet technologies the contents were primarily readable information, by which the users were limited to passive viewing, and thereby, only could retrieve information. Though, in addition the user could submit an anonymous question to an unknown healthcare professional. The questions and answers were then displayed at the website to all users. However, the website was used passively by most users, with less than 1% of the users submitting anonymous questions. This type of online helpline does not seem to respond to the need for individualized information, as the users still expressed needs for highly individualized information. Pinnock et al. [32] link the lack of individualized information to the passive role and describe how unique features such as searching, sorting, and interactivity have the capacity to respond to the need for individualized information. The evaluation of the Online Patient Book^©^ documents the importance of complementing the functionality of the static web pages by including more dynamic websites, which allow the users to interact and collaborate in a dialogue.

Furthermore, both the project by Pinnock et al. [32] and the Online Patient Book^©^ support the potential of utilising the online media even when the patient groups represent patients over 50 years. Despite that the age of the patient group, the evaluation contains several comments that the patients see health informatics tools as an important and natural way of getting information from and communicating with the healthcare professionals. Some patient users directly recommended future use of online contacts both in relation to men with prostate cancer and other group of patients.

The effects of the tool must be understood in the light of the information and communication the individual patient is offered in all. This means that the Internet based dialogue between the patient and the healthcare professionals do not stand alone. The patients in this study were also provided with the standardized oral information and opportunity for dialogues in scheduled meetings at the hospitals. At the same time, the Online Patient Book^©^ offered both monologue and dialogue-based applications, as listed in the Method section. However, this paper focuses on the patient users’ descriptions related to the asynchronous, written dialogue with the healthcare professionals at the Department of Urology.

From this research it is evident that the online dialogue in the Online Patient Book^©^ is possible and that access to it makes a difference, but maintaining and further developing health informatics tools specifically for patients will require the ongoing use of such tools in daily clinical practice and that healthcare professionals are active users. Broom [33] explains how the qualitative effects for patients who use the Internet relies on the healthcare professionals being active and positive. The Internet allows the patients to act, however, this will only be the case if healthcare professionals are aware and accept the new roles that exist between a patient who is empowered through his use of the Internet and the healthcare professionals. As Broom [33] stresses the healthcare professionals must provide encouragement, guidance, and support to patients in relation to their Internet usage to achieve the maximum benefits. Additionally, in relation to dialogue-based web applications, such as the Online Patient Book^©^, the healthcare professionals also have to take an active role in the usage of the tool to support patients.

Or et al. [34] explain how nurses felt uncomfortable using a health informatics system, if they perceived the use of it as a burden to their patients. Therefore to be an active and positive healthcare professional user it is important that the particular health informatics system improves the quality of care and treatment. Looking at the Online Patient Book^©^ it is therefore relevant to comment about how the healthcare professional users actually saw the importance of this tool. The healthcare professionals’ experiences, in this instance, points to how the Online Patient Book^©^ was of great value for the patients. The Online Patient Book^©^ therefore also became a tool for the healthcare professionals, which they indicate can contribute to increasing quality in the care and treatment of their patients, and especially in relation to meeting the needs of the individual patient. Even though these experiences were only expressed by the healthcare professionals in informal meetings, and therefore not a part of the data gathering in this evaluation, such experiences are very important to establish a positive environment around the every day use of the tool in the clinical practice. However, further evaluations are needed and planned to explore in detail the healthcare professionals’ experiences.

The relevance of dialogue-based applications, which the patients can use at home, is comparable with findings in a study by Andersen and Ruland [35]. Messages via e-mail between patients at home and nurses were analysed using content analysis. This contributed to a picture of patients with cancer who are inadequately informed even when it comes to basic knowledge in relation to their disease, symptoms, treatment, side effects, or rehabilitation. The patients had several serious unanswered questions and concerns, which could contribute to considerable uncertainty and anxiety.

The continuously open electronic door that the dialogue-based web application offers; the online, asynchronous and written environment, appears to accommodate the patients’ need to get in contact without having the feeling of disturbing the healthcare professionals, which was a problem according to the literature survey as depicted in the Background section of this paper. The advantages and importance of the asynchronous online environment is underpinned by Clemensen [36] who points out the limiting aspects when using telephone, as much time is often wasted. Minimizing waiting time is important both for the patients and for the healthcare professionals and findings in the current study document the relevance in the usage of web application of asynchronous, written contacts for that purpose.

Based on their preliminary results Ruland et al. [37] consider that online communication with nurses is important in order to reduce the patients’ unplanned consultations with doctors, as these are often the result of the unanswered questions and concerns based on insufficient information.

From the evaluation of the Online Patient Book^©^ it is not possible to conclude whether there has been any reduction in relation to readmission or telephone consultations in the first year of implementation, as the clinical practice has also been influenced by other factors such as the reduction of admission time and changed techniques and procedures in relation to anaesthesia, surgery, and so on. However, it is intended that future study will explore whether quantitative effects are also present.

Both Andersen and Ruland [35] and Moore and Sherwin [15] point out the initial fears the healthcare professionals have for inappropriate use, or an overwhelming amount, of online messages from patients. Their findings are based on analysis of the content in e-mails, however both studies initially reject these concerns. Based on the data from the patients’ evaluation of the Online Patient Book^©^, the current study confirms this rejection. The expressions in the evaluation contribute to a picture of patients who use the online contact because they need it in their difficult and insecure situation. The asynchronous environment contributes to a kind of freedom, an open door, which the patient can use when he needs. However, it is also evident that the patients act as respectful partners in a dialogue, including paying attention to the other participants’ time, for example by trying to avoid disturbing the healthcare professionals. This is consistent with the findings in an interview study by Dickerson et al. [38]. They describe how men who used the Internet have a more proactive attitude including a collaborative approach to patient care. The Internet helps the men to stay in control, to be an active partner and problem solver in their own course of treatment. These men seek an open communication with the healthcare professionals and will come to their consultations prepared, organizing their questions efficiently, as they realize that they need to respect the healthcare professionals time [38].

In dialogues the presence of at least two partners is a prerequisite, however, it is possible to be present despite physical separation, as presence does not depend solely on physical attendance. Instead the important aspects are to be aware of, listening to, and respond to the other party [22,39,40]. In relation to the online environment this means to be aware of, listen to, and respond to the written contributions. Thus, in online asynchronous and written environments, as utilized in this intervention study, reciprocity is significant. All postings have to be responded to as it signals presence and awareness. In the evaluation of the Online Patient Book^©^ the patient users were asked directly whether they experienced any periods when they did not receive an answer within 24 hours. Three patient users responded with an affirmative answer. One patient user contacted the helpdesk following a weekend when he did not get an answer. Whether this neglect was due to workload or oversight is not clear, however, the healthcare professionals still experienced the 24 hours as significant and so, their daily use was supported by an action card placed at a central point in the departments.

### Limitations

There were a number of limitations to the current study. Of the eligible participants 59 percent responded to the invitation to evaluate. It is possible that the patients who valued the Online Patient Book^©^ were more likely to undertake the evaluation. However, none of the patient users stated that the tool had a negative effect on their contact with the healthcare professionals. As the Online Patient Book^©^ is still a part of the standard care in clinical practice it is both possible and relevant to supplement the current evaluation with face-to-face interviews, content analysis of the dialogues generated, and quantitative methods to document reduced resource consumption and with other groups of responders.

The Internet application for online dialogue between the individual patient and healthcare professionals involved in his care is integrated in the health informatics tool: The Online Patient Book^©^. It is a tool that is designed to be the patients’ health informatics tool, however with a focus particularly on men with prostate cancer treated with prostatectomy surgery. The tool, in its current version, is therefore only relevant in this specified context and for this specific group of patients. However, the Online Patient Book^©^, as a model, has gain interest in other contexts in Denmark both in relation to other patient groups and other hospital settings. The future use of online contacts, both in relation to men with prostate cancer and other group of patients, is underpinned by the direct recommendation from patient users in the current study. However, further development of the Online Patient Book^©^ to other patient groups or hospital settings will require involvement of patients and healthcare professionals in these contexts. Only through the involvement of these new groups of users, can a relevant patients’ health informatics tool for other patient groups be developed.

In addition to these aspects both structural and cultural issues must be considered in relation to future use of systems as the Online Patient Book^©^ internationally. The development of the Online Patient Book^©^ took place in Denmark influenced by structural components [41] and cultural issues [42,43] present in this nation. As with any other health information systems these issues should be taken into account when considering technology transfer. In the case of the Online Patient Book^©^ the basic premise is a sufficient and established infrastructure. The patients and the hospitals must be online with reliable access to the Internet, but the staff should also be granted the possibility to communicate with the patients within a given timeframe. In some health systems each individual service is subject to payment, and in these cases the service delivered by online communication with patients must be considered.

In Denmark personalized online registries have existed for many years, as the unique personal identifier was introduced in 1968. Furthermore, all the necessary legislation has long been in place clearing the way for secure log-on and secure transmission of sensitive communications. These issues should also be considered when transferring the system to another environment.

Issues of liability may also vary from country to country, but the alteration from traditional synchronous audio telephone communication to asynchronous written communication should not pose any further complications. As when the healthcare professionals recognise any ambiguities in their contact with the patients, for example in the telephone contact, they will always ask to see the patient in person at the hospital.

Reflecting these limitation, the Online Patient Book^©^ represents an example of a health informatics system that primarily aimed to meet the information and communication needs of the patients and could therefore be a basis-model for future design processes of patients’ health informatics tools.

## Conclusion

It is substantiated that an online contact between the individual patient and the healthcare professionals can contribute to accessibility as the patients experience the healthcare professionals as easy available via the asynchronous environment. At the same time, the written online environment contributes to exchangeability as the patients and the healthcare professionals are offered a room to exchange information, questions, and answers. Thereby the dialogue-based Internet technology contributes to the two aspects that are the basis for dialogues that can accommodate the individual patient’s information and communication needs.

The online asynchronous written environment seems to add new possibilities beneficial to the patients by differentiating and expanding the opportunities for contacts outside the few scheduled face-to-face hospital contacts. The patients emphasize the feelings of having freedom from the hospital system whilst at the same time feeling secure, as they know that the healthcare professionals are only a move, on their own computer, away from them and at all times. The patients experience the online contact as a promotion to an active partner in the dialogue, as well as the easy access to relevant information empowers them significant.

Controversially, this research has indicated that in effect the preconception among healthcare professionals that when patients ask a question they want immediate responses may be false. In fact the findings of this study illustrate how the patients actually saw some advantages in not having the answers at the same minute as the question was asked. To patients, the home context was a calmer environment to ask the question in, by which they experience that their questions were more qualified. At the same time, the patients describe that they experience the answers given from the healthcare professionals as competent. The healthcare professionals also indicated that there is room for the provision of more qualified answers through this method. At informal meetings they describe how they, as healthcare professionals, utilized the flexibility in the asynchronous contact, for example by consulting each other before answering a question, as well as utilize the calmest time during their workday to respond to the patients’ questions.

So instead of seeing the introduction of online contacts as a way of decreasing the healthcare services, offering applications for asynchronous, written dialogues can be recognized as increasing the healthcare service. Utilizing the asynchronous written environment the Internet technologies offer, the patients gain freedom to continue living their life, and “visit” the hospital in times of needed and without waiting time.

## Competing interests

The authors associated with this study have no competing interests.

## Authors’ contribution

CDB have made substantial contributions to conception and design, acquisition of data, and analysis and interpretation of data, including drafting the manuscript. BSL have been involved in drafting the manuscript and revising it critically for important intellectual content including given final approval of the version to be published. CD have been involved in drafting the manuscript and revising it critically for important intellectual content including given final approval of the version to be published. EC have been involved in drafting the manuscript and revising it critically for important intellectual content including given final approval of the version to be published. EC has provided assistance with the English language. CN have been involved in drafting the manuscript and revising it critically for important intellectual content including given final approval of the version to be published. All authors read and approved of the final version of the manuscript.

## Pre-publication history

The pre-publication history for this paper can be accessed here:

http://www.biomedcentral.com/1472-6947/12/96/prepub
